# Preterm Birth Risk and Maternal Nativity, Ethnicity, and Race

**DOI:** 10.1001/jamanetworkopen.2024.3194

**Published:** 2024-03-21

**Authors:** Alejandra Barreto, Brielle Formanowski, Michelle-Marie Peña, Elizabeth G. Salazar, Sara C. Handley, Heather H. Burris, Robin Ortiz, Scott A. Lorch, Diana Montoya-Williams

**Affiliations:** 1 Department of Population and Public Health Sciences, University of Southern California, Los Angeles; 2 Division of Neonatology, The Children’s Hospital of Philadelphia, Philadelphia, Pennsylvania; 3 Division of Neonatology, Emory University School of Medicine and Children’s Healthcare of Atlanta, Atlanta, Georgia; 4 Department of Pediatrics, Perelman School of Medicine, University of Pennsylvania, Philadelphia; 5 Leonard Davis Institute, University of Pennsylvania, Philadelphia; 6 Department of Obstetrics and Gynecology, Perelman School of Medicine, University of Pennsylvania, Philadelphia; 7 Department of Pediatrics, New York University Langone Health, New York; 8 Department of Population Health, New York University Langone Health, New York; 9 Institute for Excellence in Health Equity, New York University Langone Health, New York

## Abstract

**Importance:**

Immigrant birthing people have lower rates of preterm birth compared with their US-born counterparts. This advantage and associated racial and ethnic disparities across the gestational age spectrum have not been examined nationally.

**Objective:**

To examine associations of maternal nativity, ethnicity, and race with preterm birth.

**Design, Setting, and Participants:**

This cohort study used birth certificates from the National Vital Statistics System to analyze in-hospital liveborn singleton births in the US between January 1, 2009, and December 31, 2018. Data were analyzed from January to June 2023.

**Exposure:**

Mutually exclusive nativity, ethnicity, and race subgroups were constructed using nativity (defined as US-born or non–US-born), ethnicity (defined as Hispanic or non-Hispanic), and race (defined as American Indian or Alaska Native, Asian, Black, Native Hawaiian or Other Pacific Islander, White, or other [individuals who selected other race or more than 1 race]).

**Main Outcomes and Measures:**

The primary outcome of interest was preterm birth. Modified Poisson and multinomial logistic regression models quantified relative risk (RR) of preterm birth overall (<37 weeks’ gestation) and by gestational category (late preterm: 34-36 weeks’ gestation; moderately preterm: 29-33 weeks’ gestation; and extremely preterm: <29 weeks’ gestation) for each maternal nativity, ethnicity, and race subgroup compared with the largest group, US-born non-Hispanic White (hereafter, White) birthing people. The RR of preterm birth overall and by category was also measured within each racial and ethnic group by nativity. Models were adjusted for maternal demographic and medical covariates, birth year, and birth state.

**Results:**

A total of 34 468 901 singleton live births of birthing people were analyzed, with a mean (SD) age at delivery of 28 (6) years. All nativity, ethnicity, and race subgroups had an increased adjusted risk of preterm birth compared with US-born White birthing people except for non–US-born White (adjusted RR, 0.85; 95% CI, 0.84-0.86) and Hispanic (adjusted RR, 0.98; 95% CI, 0.97-0.98) birthing people. All racially and ethnically minoritized groups had increased adjusted risks of extremely preterm birth compared with US-born White birthing people. Non–US-born individuals had a decreased risk of preterm birth within each subgroup except non-Hispanic Native Hawaiian or Other Pacific Islander individuals, in which immigrants had significantly increased risk of overall (adjusted RR, 1.07; 95% CI, 1.01-1.14), moderately (adjusted RR, 1.10; 95% CI, 0.92-1.30), and late (adjusted RR, 1.11; 95% CI, 1.02-1.22) preterm birth than their US-born counterparts.

**Conclusions and Relevance:**

Results of this cohort study suggest heterogeneity of preterm birth across maternal nativity, ethnicity, and race and gestational age categories. Understanding these patterns could aid the design of targeted preterm birth interventions and policies, especially for birthing people typically underrepresented in research.

## Key Points

**Question:**

How do the risks of extremely, moderately, and late preterm birth vary across maternal nativity, ethnicity, and race?

**Findings:**

In this cohort study of over 34 million singleton live births using US birth certificate data, there was significant variation in the associations of maternal nativity, ethnicity, and race with gestational age categories of preterm birth.

**Meaning:**

Findings of this study suggest that understanding population-wide patterns of preterm birth could aid in targeted interventions and policies, especially for birthing people underrepresented in research.

## Teaser

This cohort study uses birth certificate data to examine the risk of extremely, moderately, and late preterm birth across categories of maternal nativity, ethnicity, and race.

## Introduction

In the US, preterm birth (<37 weeks’ gestation) occurs in approximately 1 in 10 births.^1^ In 2021, preterm birth rates reached 10.5% of all live births, the greatest reported level since 2007.^1^ Preterm infants are at increased risks of mortality and lifelong impairments compared with term infants, complications that are magnified among extremely preterm infants.^2,3^ Additionally, racial and ethnic disparities in preterm birth persist and are associated with inequitable infant mortality risk.^4,5^ As of 2020, non-Hispanic Black (hereafter, Black) females and birthing people (hereafter, birthing people) continued to experience 50% higher rates of preterm birth than non-Hispanic White (hereafter, White) birthing people.^1^ Preterm birth disparities are also experienced by birthing people of other groups, particularly American Indian or Alaska Native and Native Hawaiian or Other Pacific Islander.^6,7^ Given the increasing preterm birth rates, it is important to monitor population patterns to develop interventions targeting high-risk groups.

Nativity (where an individual is born) also has important implications for preterm birth as it may serve as a proxy for citizenship, acculturation, preferred language, exposure to interpersonal discrimination, or exposure to structural xenophobia.^8^ The immigrant paradox is an epidemiologic observation suggesting that non–US-born racial and ethnic groups have better health outcomes, particularly birth outcomes, than their in-country–born counterparts despite experiencing socioeconomic and health care access–related barriers.^9,10^ However, recent literature has found heterogeneity of perinatal outcomes within immigrant communities by race, ethnicity, and country of origin.^11,12^ In addition, the intersection of a birthing person’s identities may conflict, leading to both structural and individual advantages and disadvantages that might affect preterm birth risk.^5,13,14^ Furthermore, whereas non-US nativity may be protective, Black immigrants and other birthing people from minoritized groups may experience structural and interpersonal racism, which is known to be a risk factor of adverse birth outcomes, such as preterm birth.^15^ Prior work suggests that birth disparities for racially and ethnically minoritized birthing people compared with White birthing people appear to vary both by group and by gestation, with the widest disparities observed at shortest gestation (<28 weeks).^16^ The immigrant paradox may not manifest homogeneously among groups and may conceal some groups’ risks; however, limited studies exist with large enough sample sizes to allow for disaggregation by nativity, ethnicity, and race,^12^ especially for smaller groups, such as Native Hawaiian or Other Pacific Islander people.^12,17,18^

This study used a 10-year national cohort of US births to examine associations of maternal nativity, ethnicity, and race with preterm birth. Given the potentially different causal pathways for preterm birth across different gestational ages^19,20^ and the need to better identify high-risk groups,^21^ the secondary aim was to understand the combined implications of these identities for preterm gestational age categories, including smaller racial and ethnic groups typically excluded from studies. We hypothesized that non–US-born birthing people of each race and ethnicity would have a reduced risk of overall preterm birth compared with their US-born counterparts, but this risk would vary across gestational age categories.

## Methods

This retrospective national cohort study analyzed deidentified, restricted-use National Vital Statistics System data from birth certificates that had undergone the 2003 revision.^22^ We included all in-hospital singleton live births from January 1, 2009, through December 31, 2018. We excluded records with missing maternal characteristics of interest (eg, race, ethnicity, or nativity), neonates with gestational age or birthweight that was missing or considered to be an outlier (eg, birthweight >5 SDs from the mean for gestational age and/or gestational age <20 weeks or >45 weeks), and multiples (eFigure 1 in Supplement 1). The Children's Hospital of Philadelphia Institutional Review Board deemed this study exempt from ethics review and informed consent requirement because it was not considered human participant research. We followed the Strengthening the Reporting of Observational Studies in Epidemiology (STROBE) reporting guideline.

### Variables of Interest

As previous researchers have recommended, we intentionally used the term *minoritized* instead of *minorities* to highlight the systems of power and oppression that actively marginalize and minoritize some racial and ethnic groups, which play a role in their experience of poor health outcomes,^23,24^ an approach adopted by an increasing number of health equity scholars.^25,26^ Maternal race, ethnicity, and nativity are self-reported on US birth certificates. Race options are American Indian or Alaska Native, Asian, Black, Native Hawaiian or Other Pacific Islander, White, and other (including individuals who selected other race or more than 1 race).^27^ Ethnicity is defined as Spanish, Hispanic, Latina or not. Although some people prefer other terms, such as Latinx or Latine,^28^ we used Hispanic in this study in accordance with the National Vital Statistics System.

We defined birthing people as US-born or non–US-born based on the self-reported maternal country of birth. Although some studies have defined US territory–born birthing people as non–US-born to differentiate from dyads who live and are born in the US mainland, in this study, birthing people born in the US territories were considered to be US-born to use nativity as a proxy for health implications of citizenship rather than of geographic location of birth.^15^ Birthing people were categorized according to their composite nativity, ethnicity, and race, initially leading to 22 mutually exclusive groups (eFigure 2 in Supplement 1). All birthing people who identified as Hispanic were condensed into a category encompassing all races. There is diversity within the Hispanic community, and Hispanic experiences and health outcomes vary by race; however, intra-Hispanic racial variation was beyond the scope of this study. The following nativity, ethnicity, and race subgroups were analyzed: non–US-born and US-born Hispanic, non-Hispanic American Indian or Alaska Native (hereafter, American Indian or Alaska Native), non-Hispanic Asian (hereafter, Asian), Black, non-Hispanic Native Hawaiian or Other Pacific Islander (hereafter Native Hawaiian or Other Pacific Islander), White, and non-Hispanic other (hereafter, other).^15^

The primary outcome of interest was preterm birth (<37 weeks of gestation). Gestational age on birth certificates is based on obstetric estimates using all available data (ultrasonography and/or last menstrual period).^29^ Preterm birth was further categorized as extremely preterm (<29 weeks), moderately preterm (29-33 weeks), and late preterm (34-36 weeks).^16^

Due to known associations with preterm birth, the following maternal sociodemographic and medical covariates were included in the analysis: age, insurance type, educational level, prenatal care, diabetes and hypertension (diagnosed before or during pregnancy), and tobacco use.^30,31,32,33,34^ A binary indicator adjusted for the presence of congenital anomalies in newborns. Missing values for covariates were included as missing indicators.^35^

### Statistical Analysis

Descriptive statistics summarized and compared newborn and maternal characteristics using χ^2^ analyses. The incidence of preterm birth overall and by gestational age category was described for each nativity, ethnicity, and race subgroup. For the primary analyses, we assessed the risk of preterm birth for each nativity, ethnicity, and race group compared with US-born White birthing people. This group was chosen as the reference because its large cohort size provided statistical power to detect disparities for minoritized groups that are usually excluded or relegated to the other category. This reference choice allowed for the documentation of preterm birth disparities for all groups (compared with the White population) that is currently used by the US Census. Additionally, using the White birthing people group as the reference allowed us to document how nativity affects known preterm birth disparities.

Multivariable modified Poisson models were constructed unadjusted and were then adjusted in 2 stages. The first set of adjusted models included maternal sociodemographic covariates as well as birth state and birth year as fixed effects to account for unmeasured confounders that vary by state and time. In the fully adjusted models, we also included maternal medical risk covariates (history of tobacco use, hypertension, and diabetes) that are downstream biological risk factors for preterm birth, which have been suggested as being on the causal pathway to preterm birth disparities.^5^ Postestimation linear comparisons were conducted after fully adjusted models to identify the relative risk (RR) of preterm birth by nativity within each group. For example, preterm birth rates of US-born Asian birthing people were compared with those of non–US-born Asian birthing people. These comparisons used Bonferroni-adjusted CIs to account for multiple comparisons.

In secondary analyses, multinomial logistic regression models examined each nativity, ethnicity, and race subgroup’s risk for extremely preterm birth and moderately or late preterm birth using US-born White birthing people as the reference group. As with preterm birth overall, the risk of each preterm birth category was assessed by nativity within each group, with Bonferroni-adjusted CIs. Non–US-born American Indian or Alaska Native birthing people were included in the non–US-born other category for these analyses due to small cell sizes within each preterm birth category.

All significance tests conducted were 2-tailed, with α = .05 unless Bonferroni correction was noted otherwise. These data were analyzed from January to June 2023 using Stata, version 17 (StataCorp LLC).

## Results

The study included 34 468 901 singleton live births, and the mean (SD) maternal age at delivery was 28 (6) years. Compared with non–US-born groups, all US-born groups had a greater proportion of births among younger individuals (Table 1). Educational differences by nativity varied by group. For example, a greater proportion of non–US-born Hispanic (45.1% vs 20.6%), Asian (8.4% vs 3.7%), and Native Hawaiian or Other Pacific Islander (20.7% vs 11.6%) birthing people reported no high school diploma than their US-born counterparts, whereas the reverse was seen among White, Black, and other people. Among non–US-born Hispanic birthing people, 19.8% were not insured by either Medicaid or private insurance (and thus were potentially uninsured), whereas only approximately 10.0% of non–US-born White and Asian birthing people were in that category. Maternal hypertension was more prevalent in all US-born groups compared with their non–US-born counterparts, whereas rates of maternal diabetes were greater among all non–US-born subgroups.

**Table 1.** Demographic and Medical Characteristics by Maternal Nativity, Ethnicity, and Race From 2009 to 2018 (N = 34 468 901)^a^

Characteristic	Hispanic, No. (%) (n = 8 415 593)		Non-Hispanic, No. (%)											
			American Indian or Alaska Native (n = 272 319)		Asian (n = 2 099 546)		Black (n = 4 806 185)		Native Hawaiian or Other Pacific Islander (n = 84 444)		Other (n = 667 802)^b^		White (n = 18 123 012)	
	Non–US-born	US-born	Non–US-born	US-born	Non–US-born	US-born	Non–US-born	US-born	Non–US-born	US-born	Non–US-born	US-born	Non–US-born	US-born
No. of birthing people	4 300 179 (51.1)	4 115 414 (48.9)	3436 (1.3)	268 883 (98.7)	363 607 (82.7)	1 735 939 (17.3)	748 617 (15.6)	4 057 568 (84.4)	52 256 (61.9)	32 188 (38.1)	69 179 (10.4)	598 623 (89.6)	1 143 160 (6.3)	16 979 852 (93.7)
Age, y														
≤19	260 838 (6.1)	605 954 (14.7)	208 (6.1)	33 238 (12.4)	10 608 (0.6)	12 994 (3.6)	14 371 (1.9)	487 993 (12.0)	2793 (5.3)	2428 (7.5)	1695 (2.5)	72 308 (12.1)	16 211 (1.4)	885 185 (5.2)
20-34	3 191 846 (74.2)	3 143 325 (76.4)	2599 (75.6)	210 241 (78.2)	1 257 930 (72.5)	268 036 (73.7)	530 294 (70.8)	3 166 534 (78.0)	41 603 (79.6)	26 002 (80.8)	51 556 (74.5)	459 789 (76.8)	826 276 (72.3)	13 517 664 (79.6)
≥35	847 495 (19.7)	366 135 (8.9)	629 (18.3)	25 404 (9.5)	467 401 (26.9)	82 577 (22.7)	203 952 (27.2)	403 041 (9.9)	7860 (15.0)	3758 (11.7)	15 928 (23.0)	66 526 (11.1)	300 673 (26.3)	2 577 003 (15.2)
Educational level														
<High school	1 938 825 (45.1)	849 448 (20.6)	670 (19.5)	62 562 (23.3)	145 717 (8.4)	13 482 (3.7)	111 323 (14.9)	700 967 (17.3)	10 802 (20.7)	3727 (11.6)	6462 (9.3)	81 006 (13.5)	85 136 (7.5)	1 414 799 (8.3)
High school diploma	1 196 471 (27.8)	1 349 472 (32.8)	848 (24.7)	95 688 (35.6)	224 710 (12.9)	43 020 (11.8)	191 735 (25.6)	1 410 584 (34.8)	15 887 (30.4)	11 175 (34.7)	12 495 (18.1)	155 590 (26.0)	194 949 (17.1)	3 710 410 (21.9)
Any college	643 515 (15.0)	1 325 731 (32.2)	991 (28.8)	84 513 (31.4)	285 376 (16.4)	81 289 (22.4)	210 627 (28.1)	1 377 163 (33.9)	12 813 (24.5)	10 333 (32.1)	19 484 (28.2)	210 043 (35.1)	261 938 (22.9)	5 212 366 (30.7)
Bachelor’s degree	308 923 (7.2)	377 213 (9.2)	498 (14.5)	15 454 (5.8)	576 171 (33.2)	113 256 (31.2)	142 674 (19.1)	342 168 (8.4)	2718 (5.2)	2770 (8.6)	18 337 (26.5)	79 845 (13.3)	332 091 (29.1)	4 115 922 (24.2)
Advanced degree	116 304 (2.7)	150 598 (3.7)	292 (8.5)	5566 (2.1)	450 046 (25.9)	94 683 (26.0)	69 290 (9.3)	178 141 (4.4)	688 (1.3)	888 (2.8)	10 158 (14.7)	41 652 (7.0)	241 000 (21.1)	2 264 038 (13.3)
Insurance type														
Medicaid	2 569 815 (59.8)	2 394 029 (58.2)	1595 (46.4)	172 415 (64.1)	451 457 (26.0)	75 501 (20.8)	373 077 (49.8)	2 745 374 (67.7)	23 316 (44.6)	14 855 (46.2)	22 104 (32.0)	284 595 (47.5)	360 378 (31.5)	5 226 533 (30.8)
Private	796 351 (18.5)	1 376 365 (33.4)	1188 (34.6)	48 995 (18.2)	1 072 473 (61.8)	254 619 (70.0)	239 675 (32.0)	1 024 576 (25.3)	10 958 (21.0)	10 965 (34.1)	35 679 (51.6)	234 531 (39.2)	641 902 (56.2)	10 267 029 (60.5)
Other^c^	850 026 (19.8)	273 539 (6.7)	507 (14.8)	39 388 (14.7)	171 549 (9.9)	17 417 (4.8)	112 398 (15.0)	226 145 (5.6)	8646 (16.6)	3283 (10.2)	8955 (12.9)	46 291 (7.7)	115 414 (10.1)	1 127 865 (6.6)
Any prenatal care	4 109 359 (95.6)	3 969 854 (96.5)	3284 (95.6)	256 361 (95.3)	1 686 337 (97.1)	354 182 (97.4)	704 903 (94.2)	3 797 931 (93.6)	47 669 (91.2)	30 537 (94.9)	66 201 (95.7)	575 279 (96.1)	1 103 324 (96.5)	16 527 801 (97.3)
Presence of congenital anomalies in newborn	10 863 (0.3)	10 146 (0.3)	17 (0.5)	1309 (0.5)	3393 (0.2)	782 (0.2)	1873 (0.3)	9465 (0.2)	176 (0.3)	113 (0.4)	193 (0.3)	2089 (0.4)	3095 (0.3)	66 234 (0.4)
Tobacco use	37 635 (0.9)	208 876 (5.1)	418 (12.2)	59 496 (22.1)	13 881 (0.8)	11 393 (3.1)	6073 (0.8)	380 840 (9.4)	2417 (4.6)	3017 (9.4)	3446 (5.0)	106 174 (17.7)	41 261 (3.6)	2 581 714 (15.2)
Hypertension^d^	198 484 (4.6)	236 384 (5.7)	226 (6.6)	23 943 (8.9)	62 034 (3.6)	18 721 (5.2)	53 485 (7.1)	420 141 (10.4)	3433 (6.6)	2408 (7.5)	3738 (5.4)	44 983 (7.5)	44 612 (3.9)	1 247 248 (7.4)
Diabetes^d^	329 602 (7.7)	226 410 (5.5)	361 (10.5)	25 519 (9.5)	196 354 (11.3)	31 275 (8.6)	56 364 (7.5)	208 770 (5.2)	5291 (10.1)	2665 (8.3)	5741 (8.3)	35 863 (6.0)	70 175 (6.1)	936 363 (5.5)

^a^ All χ^2^ analyses were significant at *P* < .001.

^b^ Included birthing people who selected other race or more than 1 race.

^c^ Included birthing people who used Indian Health Service, CHAMPUS/TRICARE, other government, other, and self-pay as the payment source.

^d^ Diagnosed before or during pregnancy.

Among those born in the US, Black birthing people had the greatest rate of preterm birth overall (12.1%), whereas White birthing people had the lowest rate (7.2%) (Table 2). Among non–US-born groups, Native Hawaiian or Other Pacific Islander birthing people had the highest rate of preterm birth overall (9.8%), whereas White birthing people had the lowest rate (5.5%). All US-born groups had a greater rate of preterm birth than their non–US-born counterparts. When examining preterm birth by gestational age category, we found that the immigrant paradox among non–US-born birthing individuals was evident for all groups for late and moderately preterm birth except among Native Hawaiian or Other Pacific Islander birthing people, where rates of moderately (1.8% vs 1.6%) and late (7.4% vs 6.4%) preterm birth were higher among the non–US-born vs US-born subgroups. Rates of extremely preterm birth were similar by nativity for almost all groups except among Black birthing people; US-born Black birthing people had nearly 50% greater rates of extremely preterm birth than non–US-born Black birthing people (1.1%) (Table 2).

**Table 2.** Preterm Birth Overall and by Gestational Age Categories for US Births by Maternal Nativity, Ethnicity, and Race (N = 34 468 901)^a^

Nativity, ethnicity, and race subgroup	Preterm birth, No. (%)^b^			
	Overall	Extremely^c^	Moderately^d^	Late^e^
No. of preterm births	2 728 581 (7.9)	216 612 (0.6)	489 174 (1.4)	2 022 795 (5.9)
Non–US-born Hispanic	319 483 (7.4)	23 539 (0.6)	56 225 (1.3)	239 719 (5.6)
US-born Hispanic	344 344 (8.4)	26 752 (0.7)	60 616 (1.5)	256 976 (6.2)
Non–US-born American Indian or Alaska Native^f^	314 (9.1)	NA	NA	NA
US-born American Indian or Alaska Native	25 639 (9.5)	1726 (0.6)	4603 (1.7)	19 310 (7.2)
Non–US-born Asian	116 978 (6.7)	7233 (0.4)	19 607 (1.1)	90 138 (5.2)
US-born Asian	29 369 (8.1)	1863 (0.5)	5015 (1.4)	22 491 (6.2)
Non–US-born Black	61 074 (8.2)	8158 (1.1)	12 823 (1.7)	40 093 (5.4)
US-born Black	491 133 (12.1)	63 113 (1.6)	103 000 (2.5)	325 020 (8.0)
Non–US-born Native Hawaiian or Other Pacific Islander	5114 (9.8)	340 (0.7)	933 (1.8)	3841 (7.4)
US-born Native Hawaiian or Other Pacific Islander	2789 (8.7)	236 (0.7)	500 (1.6)	2053 (6.4)
Non–US-born White	63 076 (5.5)	4008 (0.4)	10 478 (0.9)	48 590 (4.3)
US-born White	1 213 520 (7.2)	74 978 (0.4)	205 116 (1.2)	933 426 (5.5)
Non–US-born other race^g^	5045 (7.3)	390 (0.5)	1013 (1.4)	3956 (5.5)
US-born other race	50 703 (8.5)	4276 (0.7)	9245 (1.5)	37 182 (6.2)

Abbreviation: NA, not applicable.

^a^ All χ^2^ analyses were significant at *P* < .001.

^b^ <37 Weeks’ gestation.

^c^ <29 Weeks’ gestation.

^d^ 29-33 Weeks’ gestation.

^e^ 34-36 Weeks’ gestation.

^f^ Birthing people from non–US-born American Indian or Alaska Native (n = 3436) subgroup were included in the non–US-born other subgroup for the preterm severity analyses due to small cell sizes.

^g^ Included birthing people who selected other race or more than 1 race.

**Figure 1.**
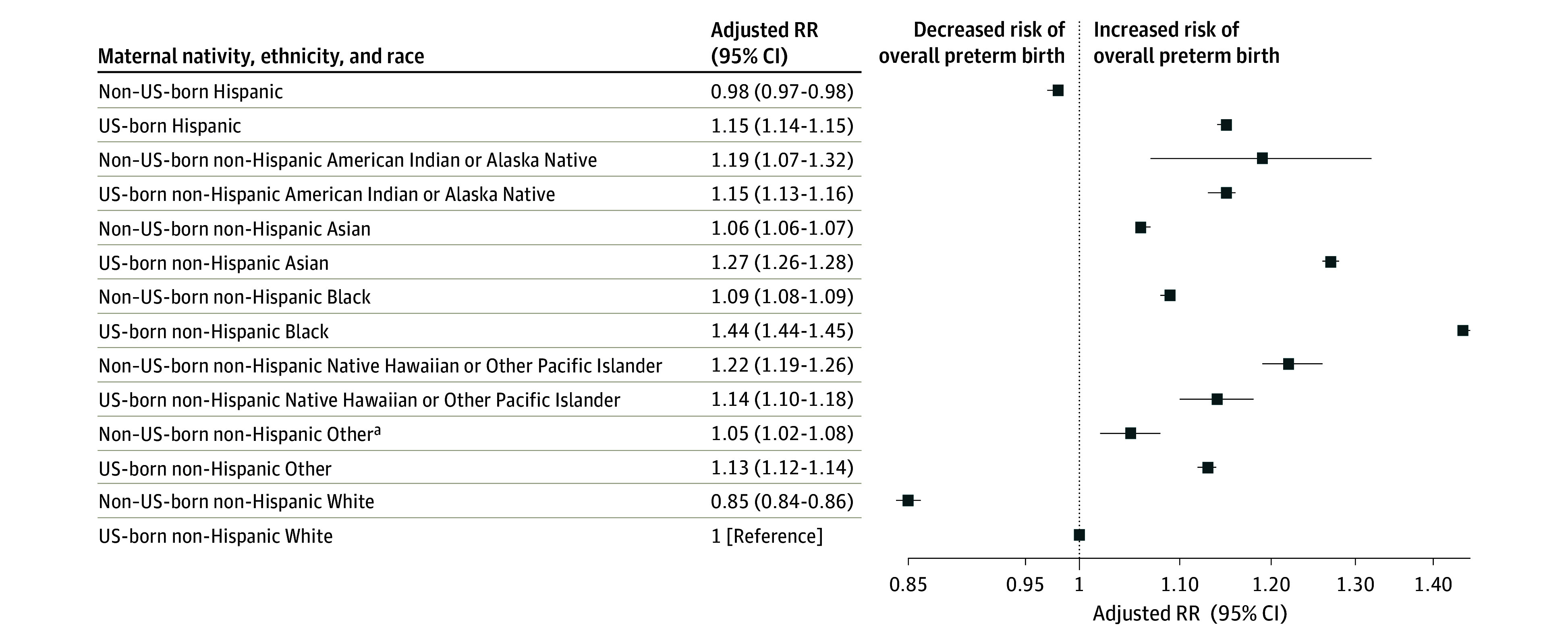
Adjusted Relative Risk (RR) of Preterm Birth by Maternal Nativity, Ethnicity, and Race All models were adjusted for maternal age, educational level, insurance type, prenatal care, tobacco use, hypertension, diabetes, presence of congenital anomalies in the newborn, birth year, and state of birth. Error bars represent 95% CIs. ^a^Included birthing people who selected other race or more than 1 race.

With respect to risk of preterm birth by maternal nativity, ethnicity, and race, all unadjusted and adjusted models are presented in the eTable in Supplement 1. Compared with US-born White birthing people, all US-born groups had a significantly increased adjusted risk of preterm birth in the fully adjusted model (Figure 1). Non–US-born White (adjusted RR, 0.85; 95% CI, 0.84-0.86) and non–US-born Hispanic (adjusted RR, 0.98; 95% CI, 0.97-0.98) birthing people were the only groups with a significantly decreased adjusted risk of overall preterm birth compared with US-born White birthing people.

**Figure 2.**
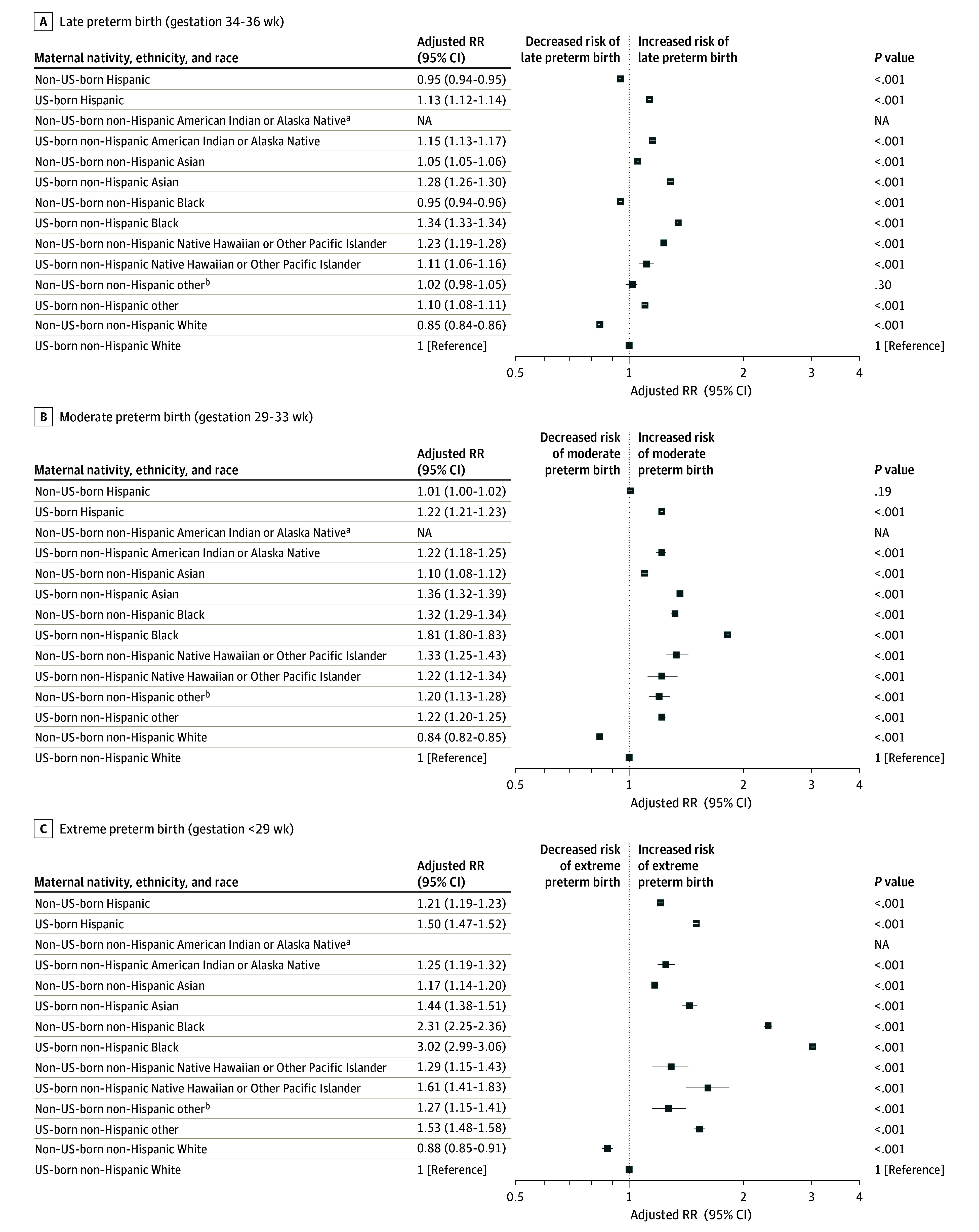
Adjusted Relative Risk (RR) of Extreme, Moderate, and Late Preterm Birth by Maternal Nativity, Ethnicity, and Race Models adjusted for maternal age, educational level, insurance type, prenatal care, tobacco use, hypertension, diabetes, presence of congenital anomalies in the newborn, birth year, and state of birth. Adjusted RRs significant at *P* < .001. Error bars represent 95% CIs; NA, not applicable. ^a^Birthing people from the non–US-born American Indian or Alaska Native (n = 3436) subgroup were included in the non–US-born other subgroup for preterm severity analyses due to small cell sizes. ^b^Included birthing people who selected other race or more than 1 race.

With respect to preterm birth gestational age categories, most nativity, ethnicity, and race subgroups had increased adjusted risk of late preterm birth compared with US-born White birthing people except for non–US-born Hispanic (adjusted RR, 0.95; 95% CI, 0.94-0.95), White (adjusted RR, 0.85; 95% CI, 0.84-0.86), and Black (adjusted RR, 0.95; 95% CI, 0.94-0.96) birthing people (Figure 2). In contrast, nearly all racially and ethnically minoritized groups had significantly increased adjusted risk of extremely and moderately preterm birth compared with US-born White birthing people except for non–US-born Hispanic people, whose risk of moderately preterm birth did not differ from US-born White people. However, the RRs of extremely preterm birth among minoritized groups compared with the US-born White group varied widely, from a mildly increased risk of extremely preterm birth among non–US-born Asian birthing people (adjusted RR, 1.17; 95% CI, 1.14-1.20) to a more substantially increased risk among US-born Black people (adjusted RR, 3.02; 95% CI, 2.99-3.06) (Figure 2).

The degree to which non–US-born birthing people had a decreased risk of preterm birth compared with their US-born counterparts varied across nativity, ethnicity, and race subgroups and gestational age categories (Table 3). Non–US-born Hispanic (adjusted RR, 0.85; 95% CI, 0.85-0.86), Asian (adjusted RR, 0.84; 95% CI, 0.82-0.85), Black (adjusted RR, 0.75; 95% CI, 0.74-0.76), and White (adjusted RR, 0.85; 95% CI, 0.84-0.86) birthing people had similarly decreased risk of preterm birth overall and across gestational age categories compared with their US-born counterparts. In contrast, non–US-born Native Hawaiian or Other Pacific Islander birthing people had a significantly increased risk of overall (adjusted RR, 1.07; 95% CI, 1.01-1.14), moderately (adjusted RR, 1.10; 95% CI, 0.92-1.30), and late (adjusted RR, 1.11; 95% CI, 1.02-1.22) preterm birth compared with US-born Native Hawaiian or Other Pacific Islander birthing people. Non–US-born American Indian or Alaska Native birthing people also had an increased risk of overall preterm birth (adjusted RR, 1.04; 95% CI, 0.90-1.20) compared with their US-born counterparts.

**Table 3.** Adjusted Relative Risk (RR) of Preterm Birth Overall and by Gestational Age Categories Among Non–US-Born and US-Born Birthing People

Nativity, ethnicity, and race subgroup	Preterm birth, adjusted RR (95% CI)^a^			
	Overall^b^	Extremely^c^	Moderately^d^	Late^e^
US-born Hispanic	1 [Reference]	1 [Reference]	1 [Reference]	1 [Reference]
Non–US-born Hispanic	0.85 (0.85-0.86)	0.81 (0.79-0.83)	0.83 (0.81-0.84)	0.84 (0.83-0.85)
US-born American Indian or Alaska Native	1 [Reference]	NA	NA	NA
Non–US-born American Indian or Alaska Native^f^	1.04 (0.90-1.20)			
US-born Asian	1 [Reference]	1 [Reference]	1 [Reference]	1 [Reference]
Non–US-born Asian	0.84 (0.82-0.85)	0.81 (0.75-0.88)	0.81 (0.77-0.85)	0.82 (0.80-0.84)
US-born Black	1 [Reference]	1 [Reference]	1 [Reference]	1 [Reference]
Non–US-born Black	0.75 (0.74-0.76)	0.76 (0.73-0.79)	0.73 (0.71-0.75)	0.71 (0.70-0.72)
US-born Native Hawaiian or Other Pacific Islander	1 [Reference]	1 [Reference]	1 [Reference]	1 [Reference]
Non–US-born Native Hawaiian or Other Pacific Islander	1.07 (1.01-1.14)	0.80 (0.62-1.04)	1.10 (0.92-1.30)	1.11 (1.02-1.22)
US-born White	1 [Reference]	1 [Reference]	1 [Reference]	1 [Reference]
Non–US-born White	0.85 (0.84-0.86)	0.88 (0.84-0.93)	0.84 (0.81-0.87)	0.84 (0.83-0.85)
US-born other race^g^	1 [Reference]	1 [Reference]	1 [Reference]	1 [Reference]
Non–US-born other race	0.93 (0.90-0.97)	0.83 (0.71-0.98)	0.98 (0.88-1.09)	0.93 (0.88-0.98)

Abbreviation: NA, not applicable.

^a^ All models adjusted for maternal age, educational level, insurance type, prenatal care, tobacco use, hypertension, diabetes, presence of congenital anomalies in newborn, birth year, and state of birth.

^b^ <37 Weeks’ gestation.

^c^ <29 Weeks’ gestation.

^d^ 29-33 Weeks’ gestation.

^e^ 34-36 Weeks’ gestation.

^f^ Birthing people from non–US-born American Indian or Alaska Native (n = 3436) subgroup were included in the non–US-born other subgroup for the preterm severity analyses due to small cell sizes.

^g^ Included birthing people who selected other race or more than 1 race.

## Discussion

This 10-year, national retrospective cohort study of maternal nativity, ethnicity, and race revealed heterogeneity in the risk of preterm birth overall and by gestational age category. Considering this intersectional maternal identity provides a more detailed understanding of racial and ethnic disparities in preterm birth that persist in the US across the gestational age spectrum. For instance, all minoritized nativity, ethnicity, and race groups had an increased risk of extremely and moderately preterm birth than US-born White birthing people; however, non–US-born Black, Hispanic, and White birthing people had a decreased risk of late preterm birth than US-born White birthing people. Although we confirmed the immigrant paradox for almost all nativity, ethnicity, and race subgroups, non–US-born Native Hawaiian or Other Pacific Islander birthing people had increased rates of overall, moderately, and late preterm birth compared with US-born Native Hawaiian or Other Pacific Islander birthing people.

These findings align with previous smaller studies that assessed racial and ethnic disparities in preterm birth among immigrants.^16,36^ Using 1995 to 2003 New York City birth certificate data, Stein et al^36^ also found an increased risk of preterm birth for both early (22-31 weeks’ gestation) and late (32-36 weeks’ gestation) preterm birth among most non–US-born Black and Hispanic groups compared with the White group. Similarly, a study using Pennsylvania births found that Black birthing people had an increased risk of extremely preterm birth compared with US-born White birthing people regardless of nativity or ethnicity.^16^

This study extends the existing literature in several key ways. First, we deepened the birth outcomes research among 2 understudied communities: American Indian or Alaska Native and Native Hawaiian or Other Pacific Islander. American Indian or Alaska Native populations experience health disparities yet are underrepresented in health, health care, and health policy–related research given their small proportion within the US population.^7^ This issue is magnified when attempting to understand health outcomes within American Indian or Alaska Native communities while acknowledging intersectional factors of risk that might be experienced by an immigrant who self-identifies as American Indian or Alaska Native. We discovered similar risks of preterm birth overall among non–US-born and US-born American Indian or Alaska Native birthing people but did not have an adequate sample size to evaluate risks across the gestational age categories for non–US-born American Indian or Alaska Native birthing people even with 10 years of national data. Some American Indian or Alaska Native birthing people choose to self-report their identity using the other category with enough frequency to change preterm birth sample-size calculations.^37^ Thus, to ensure that undercounted people receive better benefit from research and associated health care interventions, innovative ways to reclassify individuals who select the other racial and ethnic category should be explored, such as offering more detailed self-identification options on surveys or applying machine-learning techniques to evaluate write-in responses to the other category. Native Hawaiian or Other Pacific Islander populations are another group underrepresented in perinatal research^38^ and are often aggregated with Asian people, which may mask their unique outcomes given the heterogeneity within both Native Hawaiian or Other Pacific Islander and Asian subgroups.^39,40^ Native Hawaiian or Other Pacific Islander birth outcomes are important to study as they are one of the fastest-growing minoritized groups in the US,^41^ with substantial intragroup variation in perinatal outcomes.^17^

Second, we found that non–US-born Native Hawaiian or Other Pacific Islander birthing people did not experience the immigrant paradox or the expected non–US-born advantage in preterm birth. This pattern is different from what was reported in a recent analysis of a smaller dataset of US births from 2016 to 2020.^12^ The reasons for the discrepancy are unclear but may be associated with how individuals were classified in the Native Hawaiian or Other Pacific Islander category in the present cohort and what nativity represents for this group. For instance, it is unclear how people from the Pacific Islands answer the nativity question and whether this practice has changed over time, especially in former US territories, such as the Marshall Islands, which only became sovereign nations in the 1980s (during the lifetime of the birthing people in this study). Qualitative work could explore such questions to elucidate whom these epidemiologic categories represent.

Third, by examining preterm birth gestational age categories, we also shed light on the disparities in extremely preterm birth among racially and ethnically minoritized birthing communities that are not typically considered to be at an increased risk of preterm birth. For instance, the increased RRs of extremely preterm birth among US-born Hispanic and US-born Asian birthing people compared with US-born White birthing people reflect a similar disparity to the overall increased risk for preterm birth seen among US-born Black birthing people. The Asian and Hispanic communities have been frequently documented as experiencing population-level preterm birth rates similar to the White communities,^1^ which has played a role in the lack of recognition of the disparities that exist within these communities, especially in light of subgroup heterogeneity. Given that race, ethnicity, and nativity each functions as a proxy for upstream factors in disparities at both individual and structural levels,^5^ differences in preterm birth risk across the gestational age spectrum by these demographic characteristics likely indicate differential causal pathways to extremely, moderately, and late preterm birth. Without these detailed analyses, understanding of preterm birth disparities is hampered, which can limit public health efforts aimed at mitigating preterm birth.

### Limitations

This study has several limitations. Race and ethnicity data on birth certificates are highly valid and frequently used to study racial disparities in birth outcome,^42,43^ yet missing race data is a common reason for exclusion. If records with missing race are more likely to be of racially minoritized people, our assessment of disparities may be biased through undercounting. Birth certificates are also frequently used to study the implications of maternal nativity for health outcomes, but validation data are limited.^44^ Additionally, this analysis did not account for variation within Hispanic groups by race nor within any of the racial and ethnic groups by country of origin, which are known factors in intragroup variation.^11,16^ Furthermore, the analysis could not account for the length of maternal residence in the US for non–US-born individuals or for other acculturation factors in health outcomes. We also excluded out-of-hospital births, although these represent less than 2% of all births in the US.^45^ In this study, we did not account for paternal race and ethnicity due to known high missingness rates on birth certificates, which itself has been associated with poor birth outcomes.^46^

## Conclusions

This cohort study highlights the heterogeneity of the immigrant paradox of preterm birth across nativity, ethnicity, and racial identities and the gestational age. Future studies could explore how nativity, ethnicity, and race studied together may represent a way to measure the implications of intersectional structural discrimination associated with racism, colorism, and xenophobia for birth outcomes. In addition, detailed epidemiologic data such as the data presented here could aid preterm birth prevention efforts by informing the design of targeted interventions and policies to improve perinatal outcomes in specific populations.

## Notes

Supplement 1. **eFigure 1.** Flowchart of Participants Included in Analyses **eFigure 2.** Categorization of Mutually Exclusive Nativity, Ethnicity, and Racial Analytic Groups **eTable.** Unadjusted Relative Risk (RR) and Adjusted Relative Risk (aRR) of PTB, Overall and by Gestational Categories, for Women and Birthing People of Different Nativity, Ethnicity, and Race Compared to US-Born Non-Hispanic White Birthing People

Supplement 2.
Data Sharing Statement
