# Errors in Figure 2

**DOI:** 10.1001/jamanetworkopen.2024.18318

**Published:** 2024-05-20

In the Original Investigation titled “Preterm Birth Risk and Maternal Nativity, Ethnicity, and Race”^1^ published online March 21, 2024, labels in Figure 2 B and C were incorrect. This article has been corrected.
