# Supplementary material for: Preterm Birth Risk and Maternal Nativity, Ethnicity, and Race
Source: JAMA Netw Open. 2024 Mar 21;7(3):e243194. doi: 10.1001/jamanetworkopen.2024.3194 (PMC10958237; doi:10.1001/jamanetworkopen.2024.3194)
Supplement: Supplement 1. — eFigure 1. Flowchart of Participants Included in Analyses eFigure 2. Categorization of Mutually Exclusive Nativity, Ethnicity, and Racial Analytic Groups eTable. Unadjusted Relative Risk (RR) and Adjusted Relative Risk (aRR) of PTB, Overall and by Gestational Categories, for Women and Birthing People of Different Nativity, Ethnicity, and Race Compared to US-Born Non-Hispanic White Birthing People [file jamanetwopen-e243194-s001.pdf]

Supplemental Online Content

Barreto A, Formanowski B, Peña MM, et al. Preterm birth risk and maternal nativity, ethnicity, and race. *JAMA Netw Open*. 2024;7(3):e243194.
doi:10.1001/jamanetworkopen.2024.3194

eFigure 1. Flowchart of Participants Included in Analyses

eFigure 2. Categorization of Mutually Exclusive Nativity, Ethnicity, and Racial Analytic Groups

eTable. Unadjusted Relative Risk (RR) and Adjusted Relative Risk (aRR) of Preterm Birth Overall and by Gestational Categories Among Non-US-Born and US-Born Birthing People

This supplemental material has been provided by the authors to give readers additional information about their work.

© 2024 Barreto A et al. *JAMA Network Open*

eFigure 1. Flow chart of participants included in analysis

Caption: [a]Births may have more than one missing variable

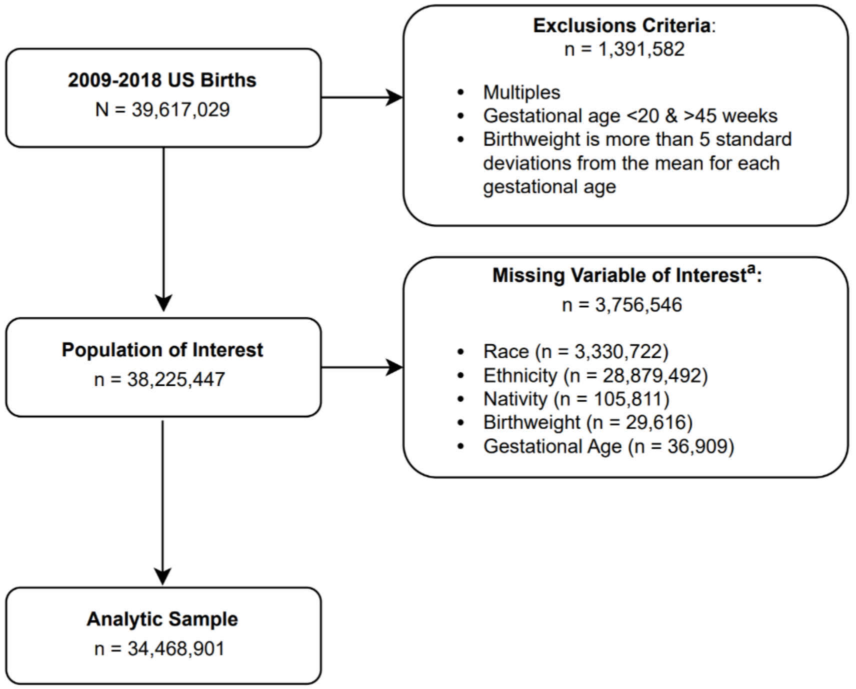

eFigure 2. Categorization of Mutually Exclusive Nativity, Ethnicity, and Racial Analytic Groups

Caption: Birthing people were initially grouped into their composite nativity, ethnic, and racial groups. All Hispanics were grouped, regardless of race to create 14 analytic groups used in the primary analysis. Non-US-born American Indian or Alaskan birthing people were included in the non-US-born other race category for the gestational categories analysis due to small cell sizes, resulting in 13 analytic groups. [a]Included birthing people who selected other race or more than 1 race.

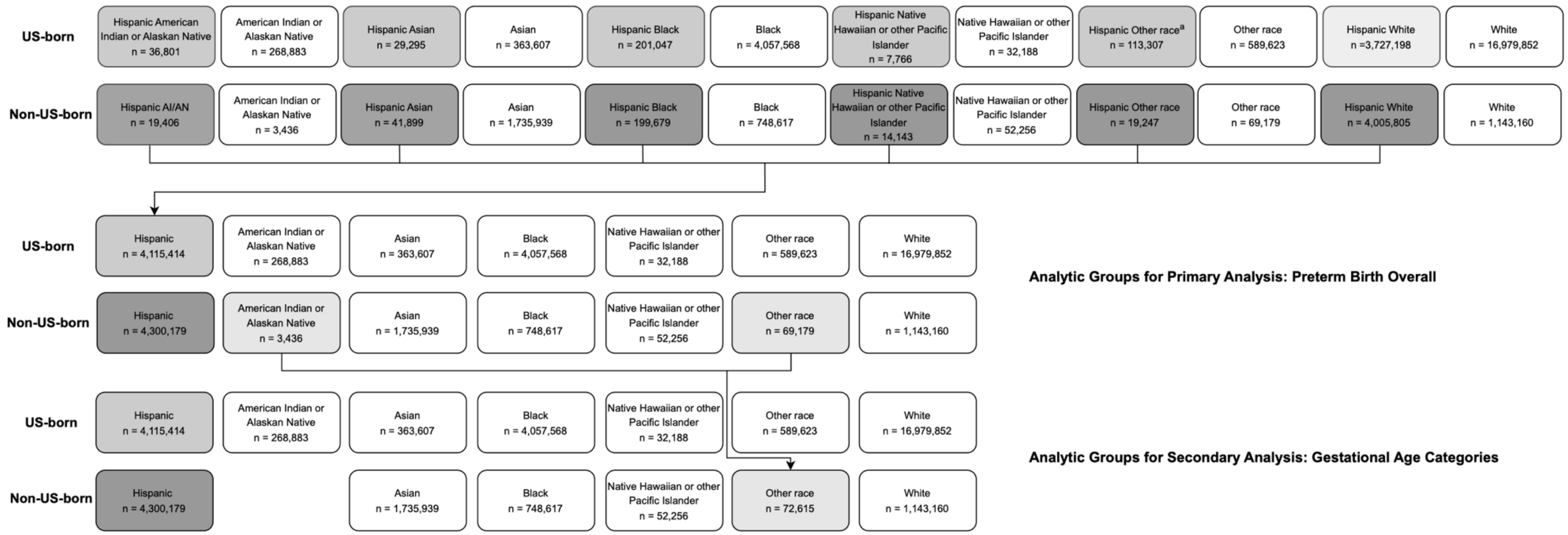

© 2024 Barreto A et al. *JAMA Network Open*

eTable: Unadjusted Relative Risk (RR) and Adjusted Relative Risk (aRR) of Preterm Birth Overall and by Gestational Categories Among Non-US-Born and US-Born Birthing People

		RR (95% CI)	aRR (95% CI)[a]	aRR (95% CI)[b]
Preterm[c]	Non-US-born Hispanic	1.04 (1.04, 1.04)	0.91 (0.91, 0.92)	0.98 (0.97, 0.98)
	US-born Hispanic	1.17 (1.17, 1.17)	1.11 (1.11, 1.12)	1.15 (1.14, 1.15)
	Non-US-born American Indian or Alaskan Native	1.28 (1.15, 1.42)	1.20 (1.08, 1.33)	1.19 (1.07, 1.32)
	US-born American Indian or Alaskan Native	1.33 (1.32, 1.35)	1.21 (1.19, 1.22)	1.15 (1.13, 1.16)
	Non-US-born Asian	1.13 (1.12, 1.14)	1.25 (1.23, 1.26)	1.27 (1.26, 1.28)
	US-born Asian	0.94 (0.94, 0.95)	1.01 (1.00, 1.01)	1.06 (1.06, 1.07)
	Non-US-born Black	1.14 (1.13, 1.15)	1.04 (1.03, 1.05)	1.09 (1.08, 1.09)
	US-born Black	1.69 (1.69, 1.70)	1.48 (1.47, 1.48)	1.44 (1.44, 1.45)
	Non-US-born Native Hawaiian or Other Pacific Islander	1.37 (1.33, 1.41)	1.20 (1.16, 1.23)	1.22 (1.19, 1.26)
	US-born Native Hawaiian or Other Pacific Islander	1.21 (1.17, 1.26)	1.16 (1.12, 1.20)	1.14 (1.10, 1.18)
	Non-US-born other race[g]	1.02 (0.99, 1.05)	1.01 (0.98, 1.04)	1.05 (1.02, 1.08)
	US-born other race	1.19 (1.18, 1.20)	1.14 (1.13, 1.15)	1.13 (1.12, 1.14)
	Non-US-born White	0.77 (0.77, 0.78)	0.79 (0.78, 0.79)	0.85 (0.84, 0.86)
	US-born White	1 [Reference]	1 [Reference]	1 [Reference]
Extremely[d]	Non-US-born Hispanic	1.24 (1.23, 1.26)	1.07 (1.05, 1.09)	1.21 (1.19, 1.23)
	US-born Hispanic	1.49 (1.47, 1.51)	1.41 (1.39, 1.43)	1.50 (1.47, 1.52)
	Non-US-born American Indian or Alaskan Native[h]	-	-	-
	US-born American Indian or Alaskan Native	1.49 (1.42, 1.57)	1.30 (1.24, 1.36)	1.25 (1.19, 1.32)
	Non-US-born Asian	0.94 (0.92, 0.96)	1.04 (1.02, 1.07)	1.17 (1.14, 1.20)
	US-born Asian	1.17 (1.12, 1.23)	1.36 (1.31, 1.43)	1.44 (1.38, 1.51)
	Non-US-born Black	2.5 (2.44, 2.55)	2.13 (2.08, 2.18)	2.31 (2.25, 2.36)
	US-born Black	3.72 (3.68, 3.76)	3.02 (2.99, 3.06)	3.02 (2.99, 3.06)
	Non-US-born Native Hawaiian or Other Pacific Islander	1.52 (1.36, 1.69)	1.21 (1.08, 1.34)	1.29 (1.15, 1.43)
	US-born Native Hawaiian or Other Pacific Islander	1.69 (1.48, 1.92)	1.58 (1.38, 1.79)	1.61 (1.41, 1.83)
	Non-US-born other race	1.21 (1.10, 1.35)	1.19 (1.07, 1.30)	1.27 (1.15, 1.41)
	US-born other race	1.64 (1.59, 1.69)	1.55 (1.50, 1.60)	1.53 (1.48, 1.58)
	Non-US-born White	0.78 (0.76, 0.81)	0.79 (0.77, 0.82)	0.88 (0.85, 0.91)
	US-born White	1 [Reference]	1 [Reference]	1 [Reference]
	Non-US-born Hispanic	1.09 (1.08, 1.10)	0.91 (0.90, 0.92)	1.01 (0.996, 1.02)
	US-born Hispanic	1.24 (1.22, 1.25)	1.16 (1.15, 1.17)	1.22 (1.21, 1.23)

© 2024 Barreto A et al. *JAMA Network Open*

**Moderately**[e]	Non-US-born American Indian or Alaskan Native	-	-	
	US-born American Indian or Alaskan Native	1.45 (1.41, 1.50)	1.30 (1.26, 1.34)	1.22 (1.18, 1.25)
	Non-US-born Asian	0.93 (0.92, 0.94)	1.00 (0.98, 1.01)	1.10 (1.08, 1.12)
	US-born Asian	1.15 (1.12, 1.19)	1.30 (1.26, 1.34)	1.36 (1.32, 1.39)
	Non-US-born Black	1.43 (1.41, 1.45)	1.24 (1.22, 1.26)	1.32 (1.29, 1.34)
	US-born Black	2.22 (2.20, 2.24)	1.87 (1.85, 1.88)	1.81 (1.80, 1.83)
	Non-US-born Native Hawaiian or Other Pacific Islander	1.52 (1.43, 1.62)	1.27 (1.19, 1.36)	1.33 (1.25, 1.43)
	US-born Native Hawaiian or Other Pacific Islander	1.31 (1.20, 1.43)	1.24 (1.14, 1.35)	1.22 (1.12, 1.34)
	Non-US-born other race	1.16 (1.09, 1.23)	1.12 (1.06, 1.20)	1.20 (1.13, 1.28)
	US-born other race	1.30 (1.27, 1.32)	1.24 (1.21, 1.26)	1.22 (1.20, 1.25)
	Non-US-born White	0.75 (0.73, 0.76)	0.75 (0.73, 0.76)	0.84 (0.82, 0.85)
	US-born White	1 [Reference]	1 [Reference]	1 [Reference]
**Late**[f]	Non-US-born Hispanic	1.02 (1.01, 1.02)	0.89 (0.88, 0.89)	0.95 (0.94, 0.95)
	US-born Hispanic	1.15 (1.15, 1.16)	1.09 (1.08, 1.10)	1.13 (1.12, 1.14)
	Non-US-born American Indian or Alaskan Native	-	-	-
	US-born American Indian or Alaskan Native	1.34 (1.32, 1.36)	1.21 (1.19, 1.23)	1.15 (1.13, 1.17)
	Non-US-born Asian	0.94 (0.93, 0.95)	1.01 (1.00, 1.02)	1.05 (1.05, 1.06)
	US-born Asian	1.14 (1.12, 1.15)	1.26 (1.24, 1.27)	1.28 (1.26, 1.30)
	Non-US-born Black	1.54 (1.53, 1.55)	1.35 (1.34, 1.36)	1.34 (1.33, 1.34)
	US-born Black	0.98 (0.97, 1.00)	0.91 (0.9, 0.92)	0.95 (0.94, 0.96)
	Non-US-born Native Hawaiian or Other Pacific Islander	1.38 (1.33, 1.42)	1.21 (1.17, 1.25)	1.23 (1.19, 1.28)
	US-born Native Hawaiian or Other Pacific Islander	1.18 (1.13, 1.23)	1.13 (1.08, 1.18)	1.11 (1.06, 1.16)
	Non-US-born other race	0.99 (0.96, 1.03)	0.98 (0.95, 1.02)	1.02 (0.98, 1.05)
	US-born other race	1.15 (1.13, 1.16)	1.11 (1.10, 1.12)	1.10 (1.08, 1.11)
	Non-US-born White	0.76 (0.75, 0.77)	0.78 (0.77, 0.79)	0.84 (0.83, 0.84)
	US-born White	1 [Reference]	1 [Reference]	1 [Reference]

[a]Models adjusted for maternal age, educational level, insurance type, prenatal care, presence of congenital anomalies in newborn, birth year, and state of birth.

[b]Models adjusted for maternal age, educational level, insurance type, prenatal care, tobacco use, hypertension, diabetes, presence of congenital anomalies in newborn, birth year, and state of birth.

[c]<37 weeks' gestation

[d]<29 weeks' gestation

[e]29-33 weeks' gestation

© 2024 Barreto A et al. *JAMA Network Open*

[f]34-36 weeks' gestation
[g]Includes birthing people who selected other race or more than 1 race.
[h]Non-US-born American Indian or Alaskan Native subgroup (N=3,436) were included in the non-US-born other race category for the preterm severity analyses due to small cell sizes.
