# Supplementary material for: Preterm Birth Risk and Maternal Nativity, Ethnicity, and Race
Source: JAMA Netw Open. 2024 Mar 21;7(3):e243194. doi: 10.1001/jamanetworkopen.2024.3194 (PMC10958237; doi:10.1001/jamanetworkopen.2024.3194)
Supplement: Supplement 2. — Data Sharing Statement [file jamanetwopen-e243194-s002.pdf]

Data Sharing Statement

Barreto. Preterm Birth Risk and Maternal Nativity, Ethnicity, and Race. *JAMA Netw Open*. Published March 21, 2024. doi:10.1001/jamanetworkopen.2024.3194

Data
Data available: No

Additional Information
Explanation for why data not available: This study examined restricted used data from the National Vital Statistics Office and thus data must be requested from them through a Data Use Agreement. For more information about analytic code and data definitions, please email the corresponding author.
